# Dynamic computed tomography assessment of patellofemoral and tibiofemoral kinematics before and after total knee arthroplasty: A pilot study

**DOI:** 10.1002/ksa.70076

**Published:** 2025-09-29

**Authors:** Miriam R. Boot, Sebastiaan A. W. van de Groes, Esther Tanck, Dennis Janssen

**Affiliations:** ^1^ Orthopaedic Research Laboratory, Department of Orthopaedics Radboud University Medical Center Nijmegen The Netherlands

**Keywords:** dynamic computed tomography, kinematics, knee, patellar tracking, total knee arthroplasty

## Abstract

**Purpose:**

To develop and evaluate the clinical feasibility of a dynamic computed tomography (CT) protocol for assessing patellofemoral (PF) and tibiofemoral (TF) kinematics before and after total knee arthroplasty (TKA), and to quantify postoperative kinematic changes in a pilot study.

**Methods:**

In this prospective single‐centre study, patients with primary osteoarthritis scheduled for cemented TKA underwent dynamic CT scans preoperatively and at 1‐year follow‐up during active flexion‐extension‐flexion. Preoperatively, the femur, tibia and patella were segmented using a neural network. Postoperatively, computer‐aided design (CAD) implant models were aligned to CT data to determine relative implant‐bone orientation. Due to metal artefacts, preoperative patella meshes were manually aligned to postoperative scans by four raters, and averaged for analysis. Anatomical coordinate systems were applied to quantify patellar flexion, tilt, proximal tip rotation, mediolateral translation and femoral condyle anterior‐posterior translation. Descriptive statistics were reported, and interoperator agreement for patellar registration was assessed using intraclass correlation coefficients (ICCs).

**Results:**

Ten patients (mean age, 65 ± 8 years; 6 men) were analysed across a shared flexion range of 14°–55°. Postoperatively, the patella showed increased flexion (median difference: 0.9°–3.9°), medial proximal tip rotation (median difference: 1.5°–6.0°), lateral tilt (median difference: 2.7°–5.5°), and lateral shift (median difference: −1.5 to −2.8 mm). The medial and lateral femoral condyles translated 2–4 mm anterior‐posteriorly during knee flexion. Interoperator agreement for patellar registration ranged from good to excellent across all parameters (ICC = 0.85–1.00).

**Conclusion:**

This pilot study demonstrates that dynamic CT enables in vivo assessment of PF and TF kinematics before and after TKA. The protocol quantified postoperative kinematic changes and demonstrated potential as research tool. Further automation is needed to investigate relationships between these kinematic patterns and patient outcomes in larger‐scale studies.

**Level of Evidence:**

Level III.

**Abbreviations**
3D: 3‐dimensional
AKP: anterior knee pain
AP: anterior‐posterior
CAD: computer‐aided design
CPD: coherent point drift
CT: computed tomography
DOF: degrees of freedom
HU: hounsfield units
ICC: intraclass correlation coefficient
IQR: interquartile range
JII‐BCS: Journey II BCS
ML: medial‐lateral
MRI: magnetic resonance imaging
PD: proximal‐distal
PF: patellofemoral
SEMAR: single‐energy metal artefact reduction
TF: tibiofemoral
TKA: total knee arthroplasty

## INTRODUCTION

Total knee arthroplasty (TKA) is a widely performed treatment for patients with end‐stage knee osteoarthritis [28]. However, approximately 20% of patients remain dissatisfied, often due to persistent anterior knee pain (AKP) and functional limitations [11, 31]. These issues have been linked to altered knee joint mechanics [4, 25, 33], yet the specific role of patellofemoral (PF) joint dysfunction remains incompletely understood [2, 32].

A major barrier to understanding post‐TKA dysfunction is the lack of a dynamic, reliable, and widely available imaging method to assess in vivo PF and tibiofemoral (TF) kinematics after surgery [17, 32]. Conventional radiographs provide only static, angle‐dependent assessments [12, 14, 17], while advanced imaging methods such as computed tomography (CT), magnetic resonance imaging (MRI) and radiography with shape‐matching offer three‐dimensional (3D) data but are restricted to discrete positions [2, 9, 13, 32]. Fluoroscopy allows dynamic evaluation, but single‐plane systems lack sufficient medial‐lateral (ML) precision [30, 32], and biplanar systems are costly and not widely available [32]. Moreover, metal artefacts from prosthetic components further complicate postoperative assessments [13].

Dynamic CT is an emerging modality that may overcome these barriers. Recent technological advancements, including wide‐detector arrays, ultrafast gantry rotation and reduced radiation exposure, have improved its feasibility in clinical settings [35]. These developments enable dynamic CT to serve as a clinically practical method for comprehensive six degrees of freedom (6‐DOF) analysis of both PF and TF kinematics.

The aim of this study was to develop and evaluate a dynamic CT‐based method for assessing PF and TF kinematics before and after TKA during an open‐kinetic chain flexion‐extension‐flexion movement. Specifically, we sought to determine the clinical feasibility of this method, and to quantify kinematics changes between pre‐ and 1‐year postoperative states. We hypothesised that this method would provide detailed, clinically relevant kinematic data that could ultimately support more personalised surgical planning and improved patient outcomes after TKA.

## METHODS

### Participants

This prospective single‐centre study was approved by the institutional review board of Radboud University Medical Centre (NL77819.091.21). All participants provided written informed consent in accordance with the Declaration of Helsinki and institutional guidelines. Between March 2022 and June 2023, patients scheduled for primary cemented TKA due to primary osteoarthritis were screened for eligibility based on predefined inclusion and exclusion criteria (Supporting Information S1: Table S1). Inclusion criteria included age between 50 and 80 years; primary noninflammatory osteoarthritis; and either neutral alignment, correctable varus deformity, or a rigid varus deformity <10°. Exclusion criteria included a body mass index exceeding 35; inability to actively extend or flex the knee; prior hip or ankle replacement surgery within the past year in the affected limb or planned hip replacement surgery within the next year. Of the 21 eligible patients, 11 consented to participate.

### CT imaging protocol

**Figure 1:**
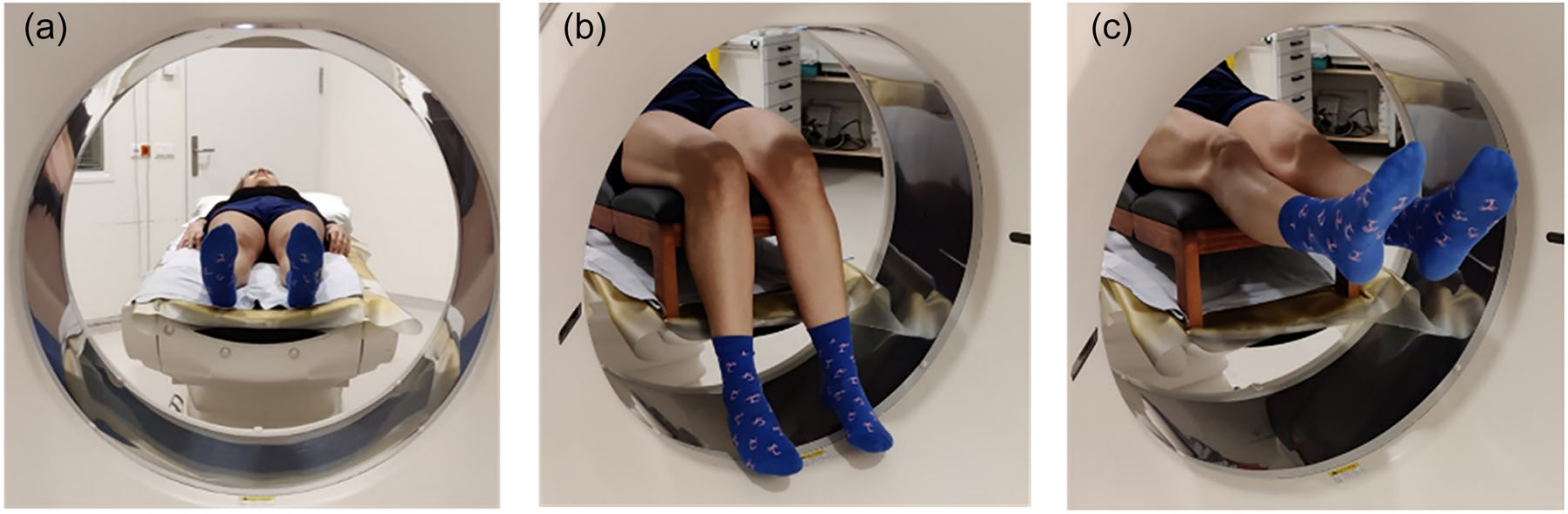
Overview of the computed tomography (CT) scanning protocol. (a) A static CT scan was performed with subjects in a supine position. (b, c) Dynamic CT scans were acquired with subjects in a semiseated position at the end of the scanner table while performing a flexion‐extension‐flexion movement from 90° to full extension with both legs simultaneously over 10 s. Adapted from Dunning et al. [8], licensed under CC BY 4.0.

Pre‐ and postoperative CT imaging was performed on a 320‐channel wide‐area CT scanner (Aquilion ONE, Canon Medical Systems). Each participant underwent static and dynamic scans both before surgery (0–6 months preoperatively) and at 1 year postoperatively to assess bone anatomy and joint kinematics. This combination of static and dynamic CT scanning allowed for precise anatomical segmentation and reliable assignment of coordinate systems, while capturing continuous joint motion for kinematic analysis. Static scans were acquired in supine position using a helical mode, with a 50 cm scan range and a mean voxel size of 0.79 × 0.79 × 0.80 mm^3^. Dynamic scans were performed with participants semi‐seated with both knees flexed to approximately 90°. Participants were instructed to perform a slow flexion‐extension‐flexion movement with both knees simultaneously over 10 s (Figure 1). After practicing, 41 dynamic CT frames were acquired with a 16 cm scan range and mean voxel size of 0.97 × 0.97 × 0.50 mm³. All scans were reconstructed using an AiCE bone kernel without single‐energy metal artefact reduction (non‐SEMAR). Preoperative acquisition parameters followed a prior study in healthy volunteers [8], whereas postoperative settings were optimised in a cadaveric study balancing metal artefacts, image quality and radiation exposure [3]. The estimated total effective radiation dose per participant was approximately 0.18 millisievert. Complete scan parameters are listed in Supporting Information S1: Table S2.

### Surgical procedure

All participants underwent bi‐cruciate stabilised cemented TKA (Journey II BCS®, Smith & Nephew Inc. [JII‐BCS]), performed by two senior orthopaedic surgeons using the CORI surgical instrumentation system. A standard medial parapatellar approach with a functional alignment technique was employed, with no ligament releases performed. Cobalt‐chromium components were chosen over oxidised zirconium to improve visualisation on CT and reduce artefacts [3]. Patellar resurfacing was initially randomised as part of a secondary research aim, but a mid‐study protocol change led to selective resurfacing based on updated manufacturer guidelines. Patellar resurfacing outcomes were not analysed in this study due to the protocol shift and small subgroup size.

### Bone and implant registration

Preoperative femur, patella and tibia were segmented from static and dynamic CT scans using a U‐Net convolutional neural network. Segmentations were converted to 3D meshes in MATLAB (R2024a, The MathWorks Inc.). Bone meshes from static CT scans were then registered to corresponding meshes from dynamic scans through point set registration using a Coherent Point Drift (CPD) algorithm [24], followed by intensity‐based B‐spline rigid image registration with the Elastix toolbox [15]. Previous research estimated the accuracy of the method at approximately 1.0° for rotational alignment and 1.0 mm for translational alignment [8].

Postoperative femoral and tibial segmentation from the static CT scans followed the same procedure. The resulting meshes were registered to their preoperative counterparts using point set registration, excluding the knee joint region to minimise errors caused by segmentation inaccuracies due to postoperative metal artefacts. Registration was refined with intensity‐based B‐spline registration with the Elastix toolbox. Due to severe metal artefacts and, to a lesser extent, motion artefacts in postoperative CT scans, automated patellar segmentation was unreliable. Instead, preoperative patellar meshes were manually aligned to postoperative scans using a custom MATLAB tool across 36 frontal, transverse and sagittal CT slices. Alignments were performed by four operators, and average positions were used for kinematic analysis to improve accuracy and reliability.

Implant positions were determined by aligning manufacturer‐provided computer‐aided design (CAD) implant models to postoperative CT scans. First, high‐intensity voxels (~2000 Hounsfield units [HU] for tibial, ~3000 HU for femoral components) were segmented via thresholding to approximate implant volumes. CAD models were coarsely aligned to these segmentations using point set registration, then refined by maximising the average grayscale intensity within the CAD‐defined implant volume using an interior‐point algorithm followed by the Nelder‐Mead simplex method [18]. Final implant positions were visually confirmed to reliably produce good alignment. The final transformation matrices, combined with known implant‐bone relationships from static CT scans, were used to determine bone positions in the postoperative dynamic CT images.

### Kinematic analyses

**Figure 2:**
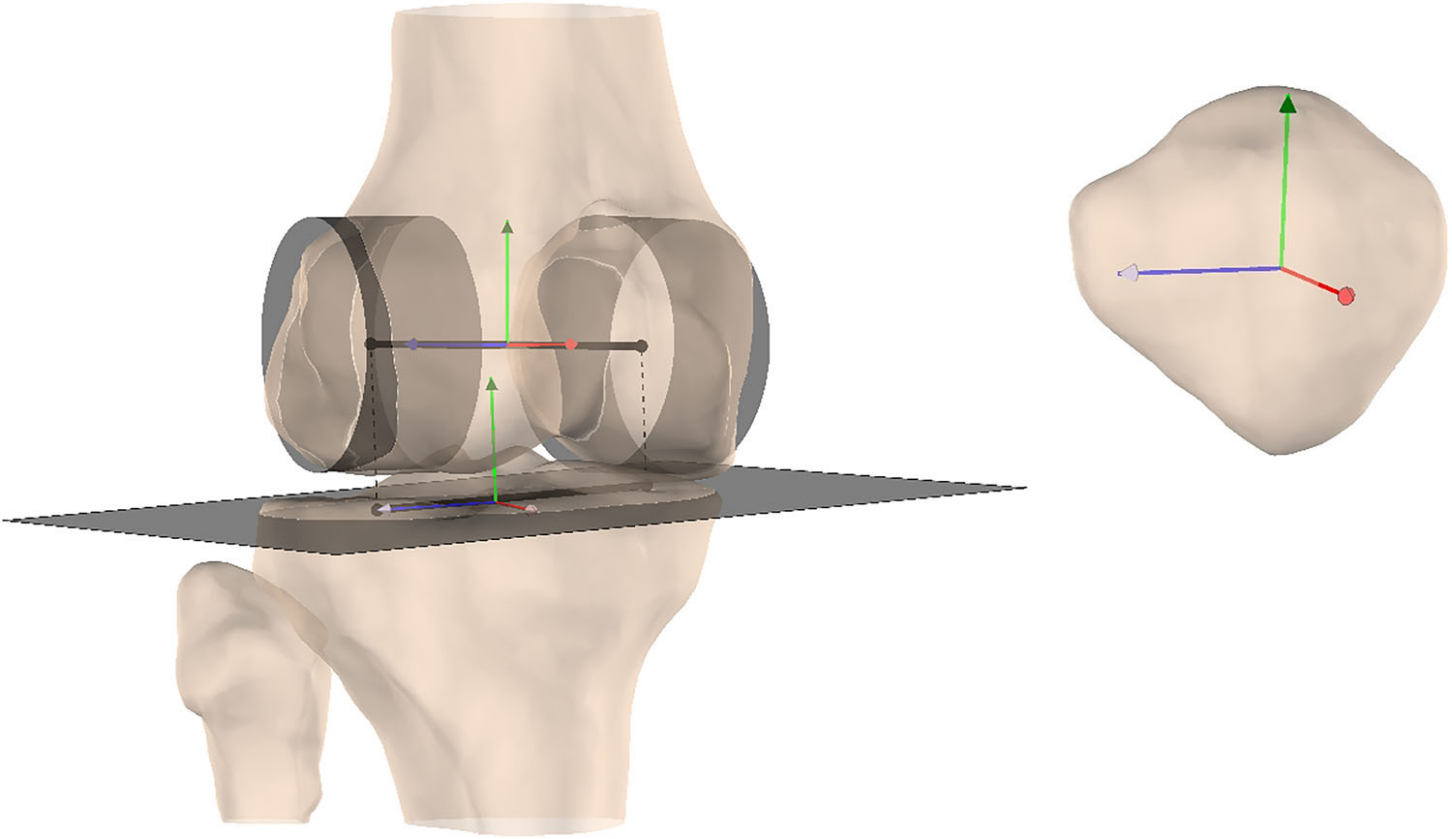
Anatomical coordinate systems of a right knee and a schematic representation of the condylar motion analysis. The definitions of coordinate systems have been previously described in detail [5, 7, 22]. Centres of cylinders fitted through the articulating surfaces of the medial and lateral femoral condyle were projected onto the transverse tibial plane to quantify anterior‐posterior (AP) translation of each condyle relative to the tibial mediolateral (ML) axis during tibiofemoral flexion.

Anatomical coordinate systems were established to define the ML, anterior‐posterior (AP) and proximal‐distal (PD) axes for the femur, tibia and patella (Figure 2), following previously validated methods [5, 7, 22]. These coordinate systems were assigned to preoperative bone meshes and transformed to align with postoperative static and dynamic CT data, enabling consistent tracking of TF and PF kinematics. PF and TF kinematics were described as the motion of the patellar and tibial coordinate systems relative to the femoral coordinate system. Rotations were determined using an intrinsic ZXY Euler sequence, with flexion/extension about the *z*‐axis, ab/adduction about the *x*‐axis, and internal/external rotation about the *y*‐axis. AP translation of the medial and lateral femoral condyles was quantified by projecting the centres of cylinders fitted to the medial and lateral condyles onto the transverse tibial plane, defined by the tibial AP and ML axes. AP translation was calculated relative to the tibial origin. PF kinematics were evaluated in terms of patellar flexion, proximal tip rotation, tilt and ML translation. These parameters were selected for clinical relevance in consultation with a senior orthopaedic surgeon (SvdG).

Analysis focused on the extension phase, reducing manual registration and computational load while retaining clinically relevant data, as previous research demonstrated that flexion and extension kinematics are highly similar in healthy knees [8]. Consequently, approximately 20 frames were analysed per participant both pre‐ and postoperatively, while postoperative patellar data were sampled at 10° TF flexion intervals to reduce manual registration time. All kinematic outputs were linearly interpolated per flexion angle to allow direct comparison across participants.

### Statistical analysis

Descriptive statistics were used to summarise participant characteristics and kinematic parameters. PF kinematics were expressed as the difference between postoperative and preoperative values (${∆}_{\text{post}-\text{pre}}$). Femoral condyle translation was reported in absolute values to compare medial and lateral patterns. Because the data were not normally distributed, results were presented as medians with interquartile range (IQR). No statistical testing was performed due to the small sample size. Interoperator agreement for manual patellar registration was assessed using intraclass correlation coefficients (ICCs) with 95% confidence intervals (CIs), based on a mean‐rating, absolute agreement, two‐way fixed‐effects model using SPSS version 27 (SPSS Inc). ICCs were interpreted according to conventional thresholds, with values between 0.75 and 0.9 considered good reliability, and values above 0.90 considered excellent reliability [16].

## RESULTS

Eleven patients were initially enrolled in this study and completed pre‐ and postoperative CT imaging. However, postoperative CT data were lost for one patient, resulting in a final dataset of ten participants (six males, four females; mean age: 65 ± 8 years; Table 1), of whom seven received patellar resurfacing during TKA. The average range of motion during the extension phase was 65° ± 9°, ranging from 55°– 81° at peak flexion to 0°–14° at full extension. This variability likely reflected unrestricted movement within the scanner, though pre‐existing flexion and extension deficits may also have contributed. To avoid bias from flexion angles reached by only a few participants, kinematic analysis was limited to the shared flexion range of 14°–55°. Clinically relevant outcome parameters are presented in the main text; additional rotational and translational data are available in the Supporting Information (Supporting Information S1: Figures S1 and S2). The total analysis time per participant was approximately 16 h. These processing times were relatively consistent across all TKA procedures, regardless of whether patellar resurfacing was performed.

**Table 1:** Participant demographics (*n* = 10).

Characteristic	Value
Age, years	64.8 ± 7.6 (53–76)
Male: female sex, *n*	6:4
Length, cm	173.7 ± 10.8 (162–192)
Weight, kg	87.9 ± 7.9 (75–105)
Body mass index	29.2 ± 2.3 (24.9–31.4)
Affected knee side; right: left, *n*	3:7
Patella resurfacing, *n*	7

*Note*: Data are shown as mean ± SD (range) or counts.

### Pre–post differences in PF kinematics

Interoperator agreement for manual patellar registration was good to excellent, with ICCs ranging from 0.95 to 1.00 for static scans and from 0.85 to 1.00 for dynamic scans (Table 2). The lowest reliability was observed for patellar tilt in the dynamic CT scans (ICC = 0.85, 95% CI: 0.78–0.90), which is still classified as good.

**Table 2:** Interoperator reliability of patellar registration in postoperative CT scans, measured by ICC and 95% CI.

Parameter	Static CT scans (*n* = 10)	Dynamic CT scans (*n *= 77*)
	ICC (95% CI)	ICC (95% CI)
Patella flexion (°)	0.98 (0.96–1.00)	1.00 (1.00–1.00)
Proximal tip rotation (°)	0.95 (0.86–0.99)	0.94 (0.90–0.96)
Patella tilt (°)	0.96 (0.89–0.99)	0.85 (0.78–0.90)
PD translation (mm)	1.00 (1.00–1.00)	1.00 (1.00–1.00)
AP translation (mm)	1.00 (1.00–1.00)	1.00 (1.00–1.00)
ML translation (mm)	0.99 (0.98–1.00)	0.97 (0.96–0.98)

*Note*: *n = 77 reflects all dynamic frames used for manual patella registration, derived from sampling at 10° tibiofemoral flexion intervals during extension.

Abbreviations: AP, anterior‐posterior; CI, confidence interval; CT, computed tomography; ICC, intraclass correlation coefficient; ML, medial‐lateral; PD, proximal‐distal.

**Figure 3:**
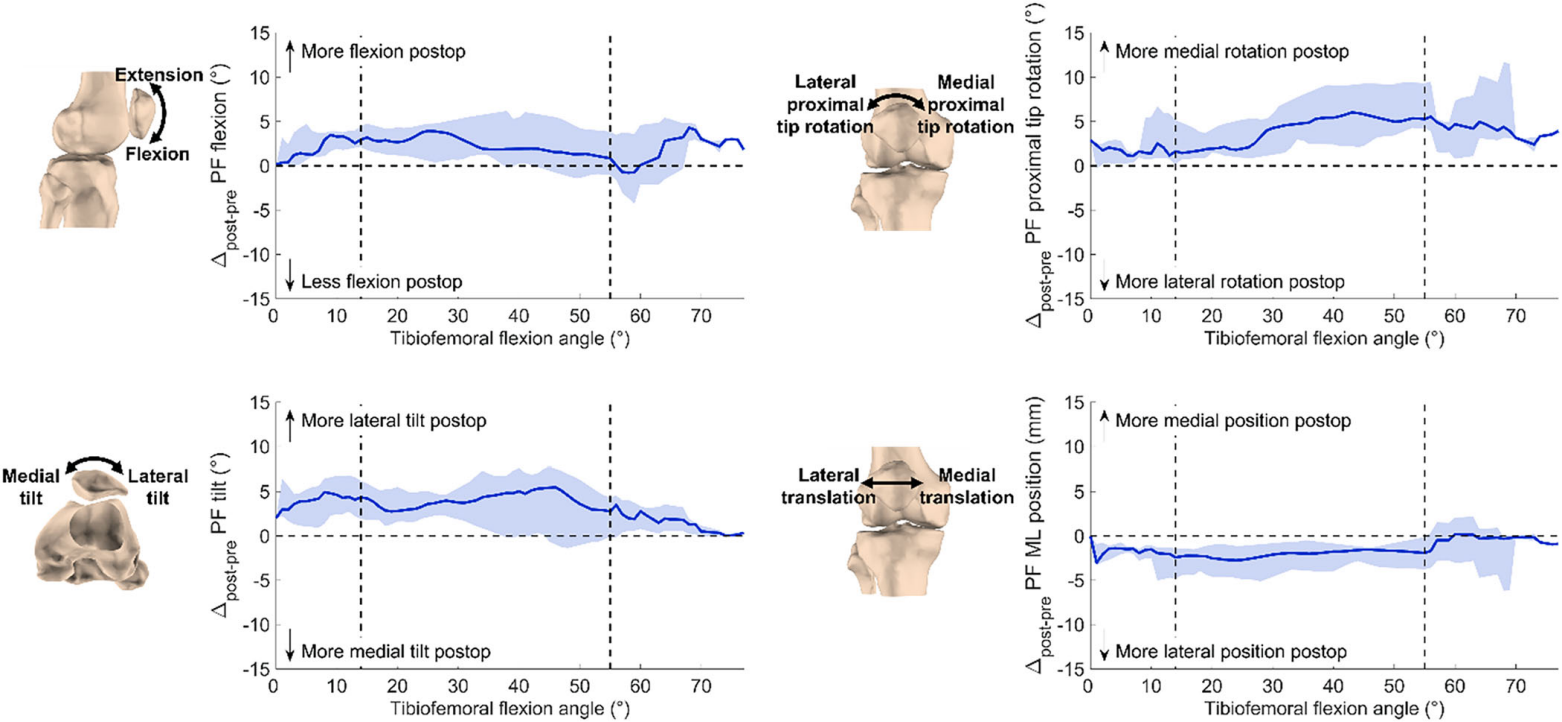
Pre‐ and post‐total knee arthroplasty (TKA) differences (${∆}_{\text{post}-\text{pre}}$) in patellofemoral (PF) flexion (top left), PF proximal tip rotation (top right), PF tilt (bottom left), and PF medial‐lateral (ML) position (bottom right) during flexion‐extension. Data are presented as median values (solid line) with interquartile ranges (shaded areas) for all 10 participants. Vertical dashed lines denote the flexion range (14°–55°) achieved by all participants pre‐ and post‐TKA; data outside this range are based on fewer participants, as not all completed the full 90°–0° extension within the scan time. Although data were obtained during knee extension, they are displayed in reverse to facilitate comparison with existing literature.

Postoperatively, the patella exhibited slightly increased flexion compared to preoperative measurements, with median changes ranging from 0.9° (IQR: −1.0 to 3.8°) to 3.9° (IQR: 0.6°–4.8°) between 14° and 55° of flexion (Figure 3). The proximal tip showed mildly increased medial rotation, with median differences from 1.5° (IQR: 0.7°–4.9°) to 6.0° (IQR: 2.2°–9.1°). Lateral patellar tilt increased by a median of 2.7° (IQR: −0.1° to 4.4°) to 5.5° (IQR: −1.0° to 7.6°), and the patella was positioned more laterally, with median changes ranging from −1.5 mm (IQR: −3.2 to −1.3 mm) to −2.8 mm (IQR: −4.8 to −1.1 mm). These directional trends were consistent across most participants. However, due to the small sample size and the absence of statistical testing, the results should be interpreted as exploratory.

### Femoral condyle AP translation

**Figure 4:**
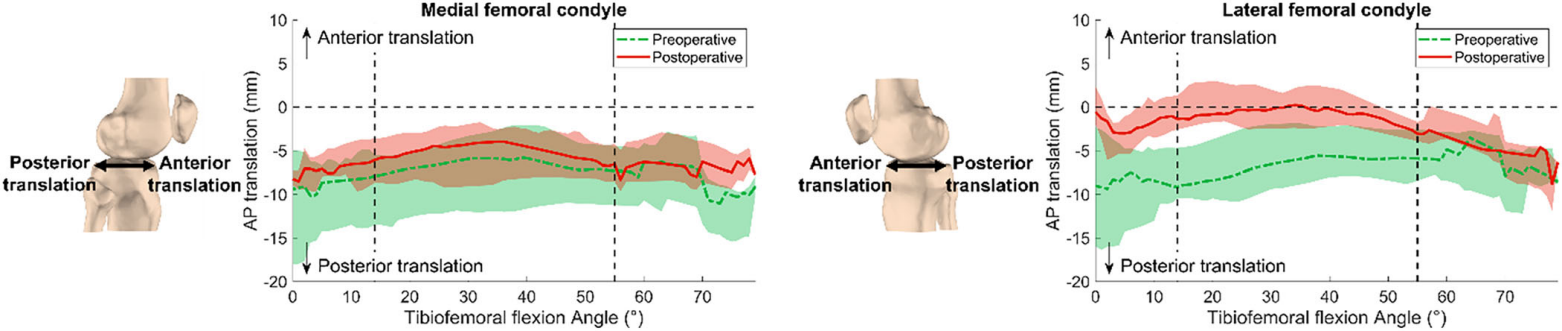
Femoral medial (left) and lateral (right) condylar anterior‐posterior (AP) translation during flexion‐extension. Pre‐TKA values are represented by green dashed lines (median) and shading (interquartile range [IQR]), and post‐TKA values by red solid lines (median) and shading (IQR). Vertical dashed lines denote the flexion range (14°–55°) achieved by all participants pre‐ and post‐TKA; data outside this range are based on fewer participants, as not all completed the full 90°–0° extension within the scan time. Although data were obtained during knee extension, they are displayed in reverse to facilitate comparison with existing literature.

The medial femoral condyle remained posterior to the AP position of the tibial origin throughout flexion both pre‐ and post‐TKA (Figure 4). Preoperatively, the medial condyle centre translated anteriorly by a median of 2.1 mm between 14° and 39° of flexion, followed by a posterior translation of 1.5 mm until 55°. Postoperatively, this pattern was preserved, with a similar anterior translation of 2.1 mm, and a slightly greater posterior translation of 2.8 mm.

The lateral femoral condyle exhibited more pronounced changes. Preoperatively, the centre translated anteriorly by a median of 3.6 mm between 14° and 38°, then stabilised. Postoperatively, the lateral centre began in a more anterior position (median at 14° of knee flexion: −1.4 mm, compared to −9.1 mm preoperatively), and showed a modest anterior shift with a median of 1.6 mm, followed by a posterior translation of 3.3 mm. Furthermore, intersubject variability in lateral condylar translation was reduced after surgery.

## DISCUSSION

This study demonstrates that dynamic CT is a promising method for continuous in vivo assessment of PF and TF kinematics before and after TKA. Key findings included postoperative trends towards increased patellar flexion, medial proximal tip rotation, lateral patellar tilt and shift, and a more anterior position of the lateral femoral condyle. To our knowledge, this is the first study to employ a dynamic CT‐based protocol for assessing post‐TKA knee kinematics, providing detailed 6‐DOF insights throughout flexion and addressing key limitations of conventional imaging techniques. Our results support its potential for broader research and clinical applications.

Postoperative PF kinematic trends included increased patellar flexion (median difference: 0.9°–3.9°), medial proximal tip rotation (median difference: 1.5°–6.0°), lateral tilt (median difference: 2.7°–5.5°), and a more lateral patellar position (median difference: −1.5 to −2.8 mm). These changes likely result from a combination of prosthesis design and alignment technique. Prosthetic trochleae, including that of the JII‐BCS, typically feature shallower grooves and reduced lateral facet height compared to the native femur [6]. These design features may reduce lateral patellar containment and contribute to increased lateral tilt and translation. Moreover, restoring native joint line obliquity with functional alignment, particularly in varus aligned knees, may have also contributed to lateralized patellar engagement. In addition to prosthesis design and alignment, component rotation [20], anatomical variation [2, 8] and methodological differences [27] may contribute to interstudy variability in PF kinematics [2, 9, 32]. Interestingly, the combination of tibial abduction, external rotation and lateral patellar shift and tilt in our cohort resembles kinematic patterns observed in healthy knees [34], which may suggest that our method is sensitive to clinically relevant variations. While patellar resurfacing was not analysed as a subgroup due to small sample size, we did not detect obvious differences in kinematic trends between resurfaced (*n* = 7) and nonresurfaced (*n* = 3) patellae on qualitative inspection.

Both medial and lateral condyles translated approximately 2–4 mm between 14° and 55° of flexion, initially shifting anteriorly, then stabilising or moving posteriorly. This pattern aligns with previous findings on condylar motion in JII‐BCS prostheses during extension [36], and may reflect a transitional phase during which neither the anterior nor posterior cam‐post mechanisms are fully engaged [10]. We did not observe a distinct lateral rollback pattern pre‐ or postoperatively, which may be due to our limited motion range [26, 36] or preoperative osteoarthritic degeneration [29]. Notably, the lateral condyle remained more anteriorly postoperatively compared to both its preoperative position and the medial condyle, suggesting increased femoral internal rotation (which is confirmed in Supporting Information S1: Figure S1, as shown by the postoperative increased external tibial rotation up to approximately 50 degrees of flexion). Additionally, reduced variability in AP translation suggests a more constrained and uniform kinematic behaviour.

Currently, no widely adopted imaging modality allows dynamic in vivo evaluation of PF kinematics in routine clinical practice [13, 17, 32]. Our dynamic CT‐based protocol has the potential to address this gap. First, CT is widely available in hospitals, and advances in wide‐detector arrays and ultrafast gantry rotation have made dynamic acquisitions more feasible [35]. Second, radiation exposure is low (0.18 millisievert), well below the annual background dose of approximately 3 millisievert [21]. Third, the method is patient‐friendly and suitable for patients with pain or limited mobility, employing a nonweightbearing, open‐chain movement that still reflects clinically relevant patterns. Fourth, using the native patellar morphology for registration enables consistent methodology for both resurfaced and nonresurfaced patellae. This flexibility is particularly useful for studies addressing the ongoing debate around the merits of patellar resurfacing [1]. Clinically, kinematic insights from dynamic CT could assist surgeons in preoperative planning by optimising component alignment to avoid excessive lateralisation, or by selecting trochlear designs with enhanced lateral containment. These strategies may help mitigate risks of AKP and joint instability. Postoperatively, the objective identification of abnormal kinematic patterns through dynamic CT could provide a clear rationale for targeted patient management, ranging from customised rehabilitation protocols to informing decisions regarding revision surgery.

This study has several limitations. First, the small sample size and use of a single implant design limit the generalisability of findings to other TKA designs and precluded statistical testing or subgroup analyses. However, the cohort was sufficient to demonstrate methodological feasibility for this implant design. Larger and sufficiently powered studies are needed to confirm these exploratory findings and assess their clinical relevance. Second, manual patellar registration was labour‐intensive, and the total analysis time per participant was substantial (~16 h), forming a barrier to clinical adoption. Future work should focus on automation and accelerating overall processing, possibly through improved image acquisition and deep learning‐based segmentation trained on artefact‐rich postoperative data. Third, the nonweightbearing protocol may not fully replicate everyday functional activities, as the absence of axial loading may influence joint behaviour [19, 23]. However, the protocol requires minimal effort, making it suitable for symptomatic patients. Future studies could include weightbearing dynamic CT protocols to better replicate functional loading conditions. Fourth, we lacked a ground‐truth reference to validate the method definitively, yet the previous research estimated the accuracy of the preoperative method at approximately 1.0° for rotational alignment and 1.0 mm for translational alignment [8]. Fifth, the analysis was limited to the 14°−55° flexion range, reflecting the range achieved by all participants, thereby precluding the evaluation of motion near full extension or deep flexion. Finally, the lack of native, predisease knee kinematics limits comparisons to pre‐ versus post‐TKA, which may not reflect healthy motion due to osteoarthritis‐induced changes [29].

## CONCLUSION

This pilot study demonstrates the feasibility of dynamic CT for in vivo assessment of PF and TF kinematics before and after TKA. The method distinguished pre‐ and postoperative kinematic patterns, revealing trends of increased patellar flexion, medial proximal tip rotation, lateral tilt and shift, and a more anterior position of the lateral femoral condyle. With further advancements in automation and processing efficiency, dynamic CT could facilitate larger‐scale investigations into the relationship between abnormal knee kinematics and patient dissatisfaction. This could ultimately guide refinements in TKA procedures, support more personalised surgical planning, and improve patient outcomes.

## AUTHOR CONTRIBUTIONS

**Miriam R. Boot**: Conceptualisation; methodology; data analysis; writing—original draft preparation. **Sebastiaan A. W. van de Groes**: Conceptualisation; methodology; data analysis; writing—review and editing. **Esther Tanck**: Writing—review and editing. **Dennis Janssen**: Conceptualisation; methodology; data analysis; writing—review and editing.

## CONFLICT OF INTEREST STATEMENT

The authors declare no conflicts of interest.

## ETHICS STATEMENT

This study was performed in line with the principles of the Declaration of Helsinki. Approval was granted by the Medical Ethical Committee of the Radboud University Medical Center (NL77819.091.21). Written informed consent was obtained from all participants (patients) in this study.

## Supporting information

Supporting information.

## Data Availability

The kinematic datasets supporting the findings of this study are openly available in the Radboud Data Repository at https://doi.org/10.34973/t28y-ys13.
