# Supplementary material for: Dynamic computed tomography assessment of patellofemoral and tibiofemoral kinematics before and after total knee arthroplasty: A pilot study
Source: Knee Surg Sports Traumatol Arthrosc. 2025 Sep 29;34(6):2087–95. doi: 10.1002/ksa.70076 (PMC13266916; doi:10.1002/ksa.70076)
Supplement: Supplementary file 1 — Supporting information. [file KSA-34-2087-s001.docx]

**DYNAMIC CT ASSESSMENT OF PATELLOFEMORAL AND TIBIOFEMORAL KINEMATICS BEFORE AND AFTER TOTAL KNEE ARTHROPLASTY: A PILOT STUDY.**

**ELECTRONIC SUPPLEMENTARY MATERIAL**

| **Inclusion Criteria** | **Exclusion Criteria** |
| --- | --- |
| Non-inflammatory knee osteoarthritis confirmed by radiology. | Valgus deformity |
| Unilateral or bilateral osteoarthritis with a functional contralateral knee, which has not been operated in last 6 months. | BMI > 35 |
| Scheduled for primary cemented total knee arthroplasty. | Recent hip/ ankle replacement surgery in the past year or planned hip joint replacement in the next year in the affected limb. |
| Aged 50–80 years, inclusive, on the day of operation. | Previous high tibial osteotomy in the affected limb |
| Stable health (ASA-score ≤ 3) and is free of or treated for cardiac, pulmonary, hematological, or other conditions that would pose excessive operative risk. | The presence of an active local/systemic infection |
| Correctable or <10° rigid varus deformity of the knee. | Incomplete/insufficient tissue surrounding the knee |
| Able to provide informed consent. | Severe damage to the medial or collateral knee ligaments and popliteal tendon |
| Available for follow-up until 2 years post-TKA. | Documented osteoporosis under active medical treatment |
| Able to walk for 2 minutes without a walking aid. | Physical/emotional/neurological conditions impacting gait, balance, or compliance with post-TKA rehabilitation and follow-up |
|  | Compromised bone quality caused by disease or infection which cannot provide adequate support and/or fixation to the prosthesis |
|  | Knee flexion < 90° |
|  | Extension deficit > 30° |
|  | Non-functional patella tendon on the affected knee, measured as an inability to actively extend the knee |
|  | Active rheumatoid arthritis, autoimmune disorders, immunosuppression, terminal illness |

**Supplementary Table S1** List of all in- and exclusion criteria

**Supplementary Table S2** Preoperative and postoperative CT scanning parameters for static and dynamic CT scans.

| **Scan type** | **Scan mode** | **Collimation** | **kV** | **mA** | **Rotation time (s/r)** | **Pitch** | **Scan time (s)** | **Slice thickness (mm)** | **Slice interval (mm)** | **Time interval (s^-1^)** | **Effective dose (mSv)** | **Coversion factor** **mSv/(mGy·cm)*** | **DLP (mGy·cm)** | **Volume CTDI (mGy)** |
| --- | --- | --- | --- | --- | --- | --- | --- | --- | --- | --- | --- | --- | --- | --- |
| **Pre-TKA, static CT** | Helical | 0.5*80 | 100 | 20 | 0.275 | 0.813 | 4.8 | 1.00 | 0.80 | - | 0.005 | 0.0005 | 10.5 | 0.2 |
| **Pre-TKA, dynamic CT** | Volume | 0.5*320 | 100 | 25 | 0.275 | - | 11.275 | 0.50 | 0.50 | 0.275 | 0.058 | 0.0005 | 116.0 | 7.2 |
| **Post-TKA, static CT** | Helical | 0.5*80 | 120 | 20 | 0.275 | 0.813 | 4.8 | 1.00 | 0.80 | - | 0.010 | 0.0005 | 19.1 | 0.3 |
| **Post-TKA, dynamic CT** | Volume | 0.5*320 | 120 | 25 | 0.275 | - | 11.275 | 0.50 | 0.50 | 0.275 | 0.106 | 0.0005 | 211.2 | 13.2 |

* Effective dose was calculated using conversion coefficients published by Saltybaeva et al. [1] based on 16.5 cm scan range for a 50 cm scan range. [1] Saltybaeva N, Jafari ME, Hupfer M, Kalender WA. Estimates of effective dose for CT scans of the lower extremities. *Radiology.* 2014; **273:** 153–159

| 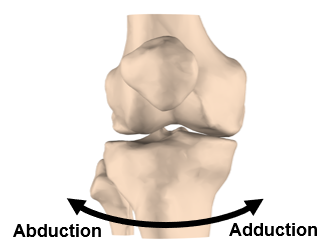  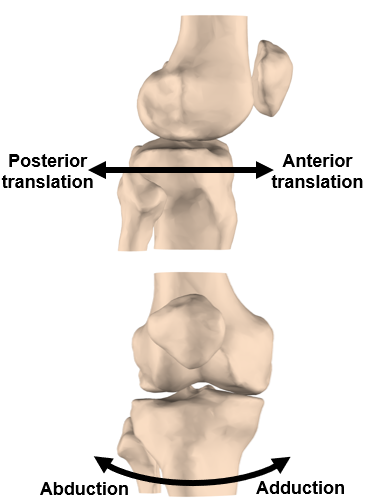 | 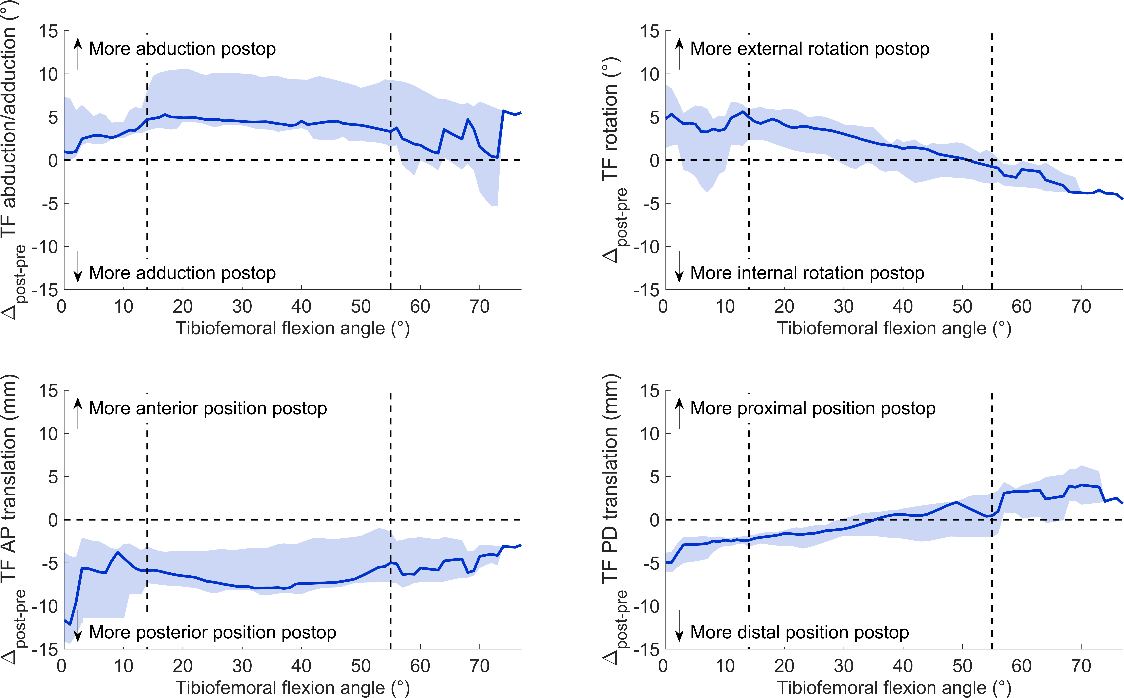 | 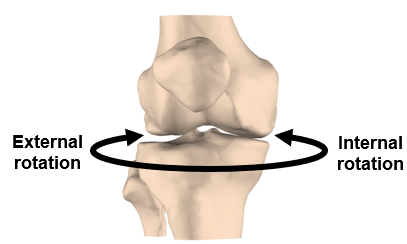  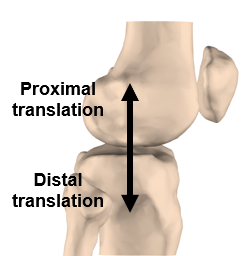 | 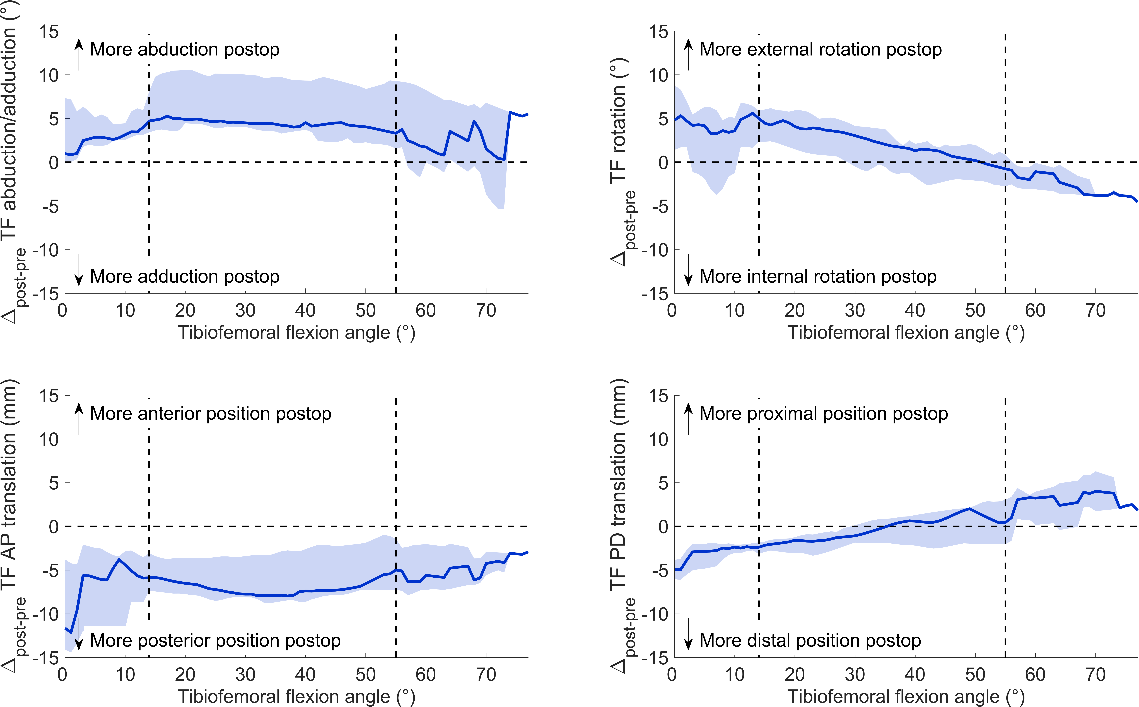 |
| --- | --- | --- | --- |
| 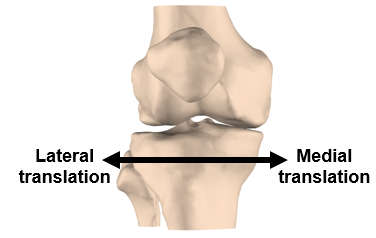 | 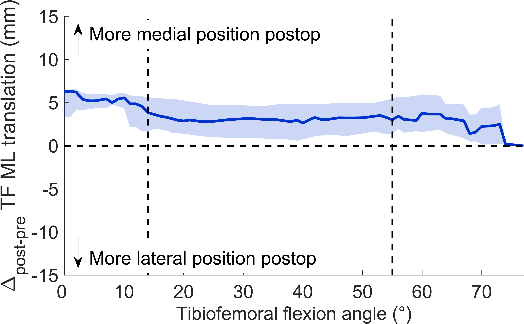 |  |  |
| **Supplementary Fig. S1** Pre- and post-TKA differences ($\Delta_{post -pre}$) in tibiofemoral (TF) abduction/adduction (top left), TF external/ internal rotation (top right), TF anterior-posterior (AP) position (middle left), TF proximal-distal (PD) position (middle right), TF medial-lateral (ML) position (bottom left) during flexion-extension. Data are presented as median values (solid line) with interquartile ranges (shaded areas) for all 10 participants. Vertical dashed lines denote the flexion range (14°–55°) achieved by all participants pre- and post-TKA; data outside this range are based on fewer participants, as not all completed the full 90°–0° extension within the scan time. Although data were obtained during knee extension, they are displayed in reverse to facilitate comparison with existing literature. | | | |

| 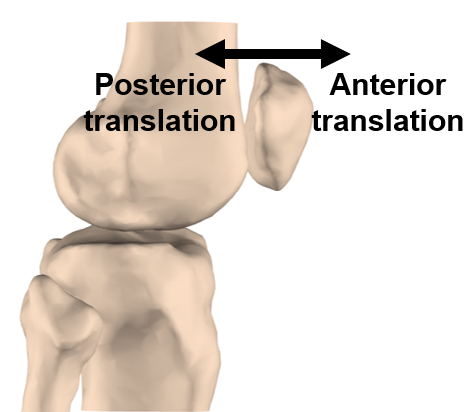 | 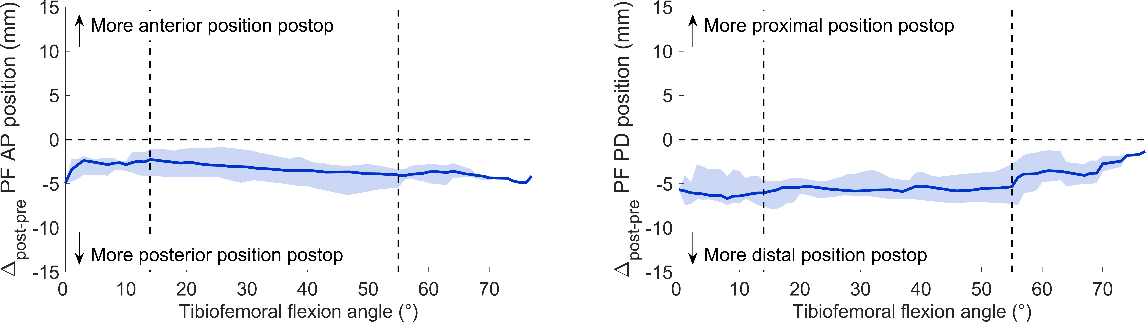 | 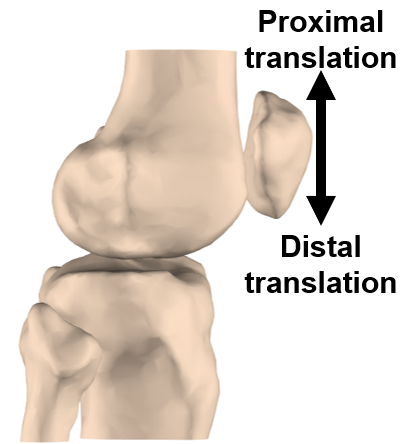 | 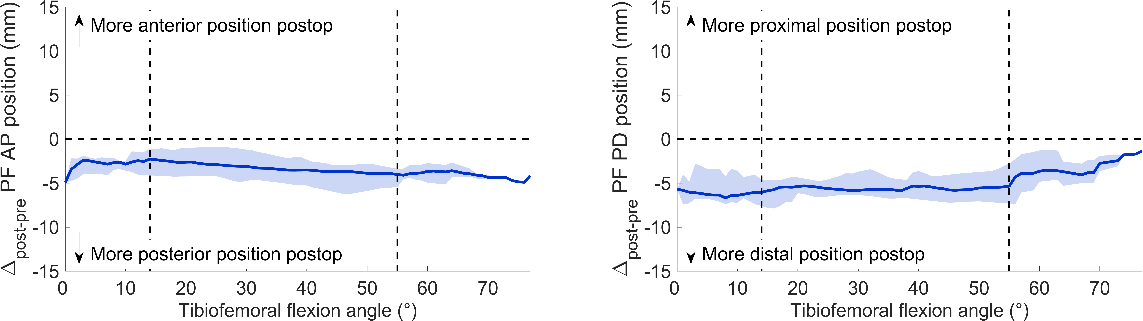 |
| --- | --- | --- | --- |
| **Supplementary Fig. S2** Pre- and post-TKA differences ($\Delta_{post -pre}$) in patellofemoral (PF) anterior-posterior (AP) position (left) and proximal-distal (PD) position (right) during flexion-extension. Data are presented as median values (solid line) with interquartile ranges (shaded areas) for all 10 participants. Vertical dashed lines denote the flexion range (14°–55°) achieved by all participants pre- and post-TKA; data outside this range are based on fewer participants, as not all completed the full 90°–0° extension within the scan time. Although data were obtained during knee extension, they are displayed in reverse to facilitate comparison with existing literature. | | | |
